# Environmental Enrichment Modulates Drug Addiction and Binge-Like Consumption of Highly Rewarding Substances: A Role for Anxiety and Compulsivity Brain Systems?

**DOI:** 10.3389/fnbeh.2018.00295

**Published:** 2018-11-29

**Authors:** Elisa Rodríguez-Ortega, Inmaculada Cubero

**Affiliations:** ^1^Departamento de Psicología, Universidad de Almería, Almería, Spain; ^2^Centro de Evaluación y Rehabilitación Neuropsicológica (CERNEP), Universidad de Almería, Almería, Spain

**Keywords:** environmental enrichment, the addiction cycle, anxiety, impulsivity/compulsivity, binge-intake

## Abstract

Drug addiction is a chronic disorder comprising components of both impulsivity and compulsivity in the so called “addiction cycle” which develops over time from early non-dependent, repetitive, binge-consumption to later post-dependent compulsive consumption. Thus, frequent binge-like intake is a typical pattern of excessive drug intake characteristic of the pre-dependent phase of the addiction cycle, which represent an important risk factor to develop addiction in vulnerable individuals. In this framework, it is of paramount interest to further understand the earliest stage of the addiction cycle so novel approaches would emerge aimed to control repetitive episodes of binge-consumption in non-dependent subjects, protecting vulnerable individuals from transition to dependence. Environmental enrichment (EE) is a preclinical animal model in which animals are housed under novel, social enriched conditions, which allows exercising and provides sensory and cognitive stimulation. EE promotes important improvements for a variety of cognitive processes and clear therapeutic and protective effects preventing ethanol (EtOH) and drug addiction as well. Interestingly, recent observations suggest that EE might additionally modulate binge-like intake of highly palatable caloric substances, including EtOH, which suggests the ability of EE to regulate consumption during the initial stage of the addiction cycle. We have proposed that EE protective and therapeutic effects on binge-consumption of palatable substances might primarily be mediated by the modulatory control that EE exerts on anxiety and impulsivity/compulsivity traits, which are all risk factors favoring transition to drug addiction.

## Introduction

Drug addiction is a chronic psychiatric disorder which exhibits components of both impulsivity and compulsivity and develops over time on three progressive stages in the so called “addiction cycle” (Koob and Volkow, 2009): (1) a binge/intoxication phase guided by the rewarding properties of drugs and impulsivity; (2) a withdrawal phase where excessive consumption escalates; and (3) a preoccupation/anticipation phase mainly guided by negative reinforcement, increased stress and anxiety and compulsivity (Koob and Volkow, 2009). Similarly to drugs, recent growing scientific evidence points to the existence of the new disorder called “food addiction” which exhibits several neurobehavioral components matching drug addiction disorders (Avena et al., 2008; Gearhardt et al., 2011; Novelle and Diéguez, 2018), and where binge eating of highly palatable/caloric substances dominates the early stages of that process (Schulte et al., 2016).

Traditionally, drug and ethanol (EtOH) addiction research is dominated by studies addressing post-dependent stages of the addiction cycle, modeled by drug/EtOH dependence (Yardley and Ray, 2016; Spanagel, 2017) and drug/EtOH relapse preclinical procedures (Marchant et al., 2013; Vengeliene et al., 2014). Because continued binge-like consumption represents a risk behavior that favors transition to addiction (Koob and Le Moal, 2006; Courtney and Polich, 2009; Crabbe et al., 2011; Thiele and Navarro, 2014), we have alternatively focused our interest during the last years in the early pre-dependent stage of the addiction cycle dominated by repetitive binge-like intake (Alcaraz-Iborra et al., 2014, 2017; Alcaraz-Iborra and Cubero, 2015; Carvajal et al., 2015; Rodríguez-Ortega et al., 2018). It is our, and others authors (Thiele and Navarro, 2014) believe, that understanding those neurobehavioral processes involved in repetitive binge consumption in non-dependent animals would help us to develop new approaches preventing transition to dependence. In this regard, we have recently addressed in our laboratory the beneficial impact of providing environmental enriched housing conditions to adolescent and adult animals showing spontaneous binge-like consumption.

It is well known that housing conditions modulate diverse psychological processes and drug addiction (Nithianantharajah and Hannan, 2006). Preclinical research have demonstrated that environmental enrichment (EE), where animals are socially grouped and exposed to sensorial and motor enriched housing conditions (Crofton et al., 2015), prevents and also modulates ongoing EtOH intake and drug addiction as well (Nithianantharajah and Hannan, 2006). Consistent with this work, we have recently extended this knowledge by showing the therapeutic and protective role of EE on excessive binge-like consumption of EtOH (Rodríguez-Ortega et al., 2018). Interestingly, there is experimental evidence indicating that EE exposure also reduces anxiety (Peña et al., 2006, 2009; Sztainberg et al., 2010; Rodríguez-Ortega et al., 2018), compulsivity (Muehlmann et al., 2012; Bechard et al., 2016; Rodríguez-Ortega et al., 2018) and modulates EtOH (Deehan et al., 2007, 2011; de Carvalho et al., 2010; Rodríguez-Ortega et al., 2018) and sucrose intake (Brenes and Fornaguera, 2008; Grimm et al., 2010, 2013, 2016). Elevated anxiety (Wand, 2005), high sensitivity to stress (Goeders, 2003) and/or high compulsivity (Figee et al., 2016) might significantly increase vulnerability to develop addiction; on the other hand, we have reported that EE modulate spontaneous binge-intake. Taking together the aforementioned data, we have proposed the working hypothesis that EE housing conditions might primarily act on anxiety and impulsivity/compulsivity related brain systems during the intoxication early phase of the addiction cycle, protecting vulnerable non-dependent organisms from excessive binge-like intake and transition to dependence (Rodríguez-Ortega et al., 2018).

In the next paragraphs we first address the impact of EE exposure on the later dependent stage of the addiction cycle and then, we focus on more recent evidence indicating the additional benefits of EE exposure on the early, binge-dominated, pre-dependent stage.

## Environmental Enrichment Exposure Regulates Drug and Sugar Intake During the Later Stage of the Addiction Cycle

### EE Protective Role in Drug Addiction

Recent studies employing animal models firmly submit that EE access during adolescence could act as a protective tool to prevent drug addiction development (Stairs and Bardo, 2009; Solinas et al., 2010). C57BL/6J mice raised under EE conditions during adolescence showed attenuated morphine-induced conditioned place preference (CPP), reduced hyperlocomotion and behavioral sensitization induced by acute morphine administration (Xu et al., 2007), and also showed blunted acute morphine-induced locomotor activity (Xu et al., 2014). Furthermore, rearing C57BL/6J mice in EE conditions reduced the reinforcing properties of heroin in a CPP test (El Rawas et al., 2009) and reduced Sprague-Dawley rats’ amphetamine self-administration (SA) in a fixed ratio (FR) paradigm (Green et al., 2002). Moreover, early EE access reduced cocaine rewarding properties in a CPP test, reduced cocaine induced motor activation, blunted responses to a repetitive cocaine challenge (Solinas et al., 2009) and decreased intravenous cocaine SA in C57BL/6J mice and Sprague-Dawley rats (Green et al., 2010).

### EE Therapeutic Role in Drug Addiction

On the other hand, there is consistent scientific evidence supporting the therapeutic effect of EE access during adulthood to modulate drug consumption, drug reward and drug relapse. Thus, in Long Evans rats trained for continued heroin administration in a FR program, EE access blunted operant responses in cue-induced reinstatement of heroin seeking (Galaj et al., 2016). In Wistar rats, exposure to EE decreased behavioral deficits induced by methamphetamine and the risk of withdrawal unleashed by relapse (Hajheidari et al., 2015). EE also reduced heroin-, nicotin- and methamphetamine-seeking responses measured by a FR schedule in Sprague-Dawley rats (Sikora et al., 2018). Additionally, cocaine-induced behavioral sensitization and CPP to cocaine were blunted in adult C57BL/6 mice exposed to EE conditions and EE also protected cocaine-pre-exposed animals from cocaine-elicited CPP reinstatement (Solinas et al., 2008). In Sprague-Dawley rats, EE access blunted cocaine and stress induced cocaine reinstatement (Chauvet et al., 2009). EE eliminated cocaine-seeking induced by context in C57BL/6 mice (Chauvet et al., 2011) and blunted the onset of cocaine craving incubation. In the same direction, EE eliminated an already stablished cocaine incubation (Chauvet et al., 2012), reduced reinstatement of cocaine operant responding elicited by a sensorial cue and also inhibited cocaine-seeking responses during extinction (Thiel et al., 2009).

### EE Therapeutic Role on Sucrose Intake and Sucrose Seeking

Interestingly, exposure to EE housing conditions do also modulate excessive consumption of highly palatable caloric substances. Isolated Sprague-Dawley rats consumed more sucrose than social or EE housed counterparts in a two bottle-choice (2BC) sucrose consumption test (Brenes and Fornaguera, 2008); isolated reared Lister hooded rats significantly drank more sucrose than socially reared rats when given sucrose in a 2BC paradigm in an ascending order of presentation (Hall et al., 1997). Additionally, single housed Wistar rats showed greater CPP for sucrose than their socially housed counterparts (Van den Berg et al., 1999). Moreover, EE access after sucrose SA training decreased sucrose seeking in Long-Evans rats, as measured by the response for a tone plus light cue previously conditioned to sucrose SA (Grimm et al., 2010). Also, acute and chronic access to EE was effective in reducing sucrose cue-reactivity and consumption in Long-Evans rats (Grimm et al., 2013, 2016). And isolated Sprague-Dawley rats were more sensitive to a cue-light compound paired with sucrose, when compared with enriched housed rats (Gill and Cain, 2011). Finally, Sprague-Dawley rats under EE housing conditions showed greater extinction of sucrose-maintained operant responding on a continuous reinforcement schedule than isolated rats (Stairs et al., 2006). Taking together, available data indicate that EE access clearly regulates sucrose consumption and sucrose seeking.

## Environmental Enrichment Exposure Regulates Binge-Like Consumption in Non-dependent Animals During the Early Stage of the Addiction Cycle

While there is consistent experimental evidence regarding the impact of EE on drug addiction, far less is known regarding the effect of EE exposure on excessive consumption of rewarding substances during early stages of the addiction cycle, dominated by binge-like intake. Preliminary data on EtOH research strongly suggest that EE exposure might provide therapeutic and protective effects to successfully modulate binge-like EtOH intake characteristic of the early stages of the addiction cycle. “Drinking in the dark” (DID; Rhodes et al., 2005, 2007) is a preclinical model of binge-like drinking which models human EtOH binge-intake as it triggers similar patterns of high voluntary EtOH consumption (BECs around 80 mg/dl) in short intervals (Cox et al., 2013; Alcaraz-Iborra et al., 2014; Thiele and Navarro, 2014; Carvajal et al., 2015). DID also models human binge consumption of caloric palatable substances. Thus, sucrose limited access on a DID procedure triggers more sucrose intake than 50% of the total amount consumed over 24 h under unlimited access (Sparta et al., 2008; Kaur et al., 2012). Given the ability of DID to model human binge-like consumption, it has been employed for studying the impact of EE on binge-like consumption of EtOH and other rewarding substances (sucrose) during the initial stages of the addiction cycle.

### EE Reduces EtOH Binge-Like Consumption

Recent evidence indicates that EE exposure might work to modulate excessive, binge-like EtOH consumption during the pre-dependent, early stage, of the addiction cycle. First, social and EE reduced EtOH preference and binge-like EtOH consumption in adult male C57BL/6 mice living in continuous (24 h) or restricted (3 h) EE conditions (Marianno et al., 2017). Also, C57BL/6 mice early socially housed showed reduced EtOH binge-like intake in a DID-2BC schedule when compared with their isolated housed counterparts (Lopez et al., 2011). EE access blunted high EtOH binge-like intake elicited by chronic social isolation in C57BL/6J mice (Lopez and Laber, 2015) and EE rearing during adolescence protected C57BL/6J mice from excessive EtOH binge-like drinking during adulthood (Rodríguez-Ortega et al., 2018). Furthermore, EE access significantly ameliorated steady EtOH binge-like consumption of adult mice housed in standard conditions (Rodríguez-Ortega et al., 2018) indicating a therapeutic role of EE on EtOH binge-like intake during that early, pre-dependent stage. Taking together, preliminary evidence points to the benefits of EE exposure, either during adolescence as a protective tool, or during adulthood as a therapeutic one, to modulate EtOH binge-like consumption in non-dependent organisms.

## Environmental Enrichment Exposure Modulates Anxiety-Like Responses and Compulsivity

High compulsivity (Figee et al., 2016), enhanced anxiety (Wand, 2005) and novelty-seeking responses (Iacono et al., 2008; Montagud-Romero et al., 2014; Arenas et al., 2016) are all premorbid neurobehavioral traits strongly linked to enhanced vulnerability to the onset of drug, food and EtOH addiction. Importantly, there is consistent scientific evidence pointing to the EE ability to regulate anxiety- and compulsivity-like behaviors as well as novelty-seeking traits. Thus, exposure to EE housing conditions reduced anxiety-like behaviors as measured by the Elevated Plus Maze (EPM) in both rats and mice (Peña et al., 2006, 2009; Sztainberg et al., 2010; Ragu Varman and Rajan, 2015; Bahi, 2017b), the Zero Maze (EZM) in rats (Nobre, 2016) and the light/dark box in mice (Sztainberg et al., 2010; Ragu Varman and Rajan, 2015). Moreover, EE housing conditions decreased motor exploration in SHR rats in an open field test (de Carvalho et al., 2010) and did blunt the onset of repetitive compulsive motor responses (Muehlmann et al., 2012; Bechard and Lewis, 2016; Bechard et al., 2016), which has been considered a manifestation of compulsivity in drug addiction (Figee et al., 2016). Finally, we have recently reported that animals under EE and long-term exposed to EtOH binge-consumption, show reduced anxiety- and compulsive-like responses, and reduced novelty-seeking behaviors, as assessed by EPM and the Hole Board (HB) test, respectively (Rodríguez-Ortega et al., 2018).

Given that, first, EE protects and ameliorates excessive EtOH binge-like drinking (Lopez et al., 2011; Lopez and Laber, 2015; Marianno et al., 2017; Rodríguez-Ortega et al., 2018), excessive sucrose consumption (Brenes and Fornaguera, 2008; Grimm et al., 2010, 2013, 2016); and second, because EE reduces anxiety (Peña et al., 2006, 2009; Sztainberg et al., 2010; Ragu Varman and Rajan, 2015; Nobre, 2016; Bahi, 2017a; Rodríguez-Ortega et al., 2018) and compulsivity-like responses (Bechard and Lewis, 2016; Bechard et al., 2016; Rodríguez-Ortega et al., 2018), we have proposed the hypothesis holding the existence of a causal nexus linking EE-mediated reduced anxiety/compulsivity and reduced EtOH binge-like consumption. Thus, we defend a primary impact of EE on anxiety/compulsivity/impulsivity brain systems during the pre-dependent early stages of the addiction cycle which, in turn might secondarily modulate binge-like intake of rewarding substances, finally preventing transition to addiction in vulnerable organisms.

## Environmental Enrichment and Human Drug Intake

Consistent with preclinical results, human research has suggested that EE exposure during the early and later, stages of the addiction cycle might be determinant to control excessive drug intake and prevent drug relapse. In this regard, community programs such as the Strategic Prevention Framework (SPF), has successfully controlled elevated EtOH binge drinking in adolescents and enforcement outcomes overtime (Anderson-Carpenter et al., 2016). Accordingly, the importance of controlling key environmental factors to prevent repetitive EtOH binge drinking episodes among students has been highlighted in national laws and drug policies, normative campaigns and targeted law enforcement (Clapp and Shillington, 2001; Clapp et al., 2003). Interestingly, programs such as Alcoholic Anonymous which encourage a controlled environment, free of drugs-cues and stress, together with enhanced social cognition and reinforcement (Schnabel, 2009), or the “Drug courts” program, which offers community-based treatment options (Turner et al., 2002), have both shown the utility of EE conditions to prevent EtOH relapse in addicted persons. Altogether, pre-clinical studies and human research, reinforce the idea that exposure to environmental enriched conditions during the different stages of the addiction cycle offer clear benefits to prevent and modulate binge-consumption, drug addiction and drug relapse.

Nevertheless, this is a young scientific field and we have a long way ahead. We need to further explore neurobehavioral and neurochemical mechanisms involved in EE positive effects, both protective and therapeutic, on the addiction cycle. We need to address whether EE exposure to non-addicted organisms impact on impulsive/compulsive consumption of also on other still non-explored drugs, during early stages of the addiction cycle, so new behavioral strategies can be developed to prevent transition to dependence. We need to further explore whether EE benefits on drug intake are primarily linked to EE-induced regulation of impulsivity/compulsivity and anxiety brain systems. We also need to understand how EE exposure during adolescence prevents during adulthood drug intake in different stages of the addiction cycle. Importantly, the role of EE on eating disorders and food addiction need to be deeply explored. In this regard, we are currently addressing in our lab whether EE exposure regulates binge-consumption of highly palatable caloric substances in non-dependent animals, so we can build testable hypothesis regarding the protector and therapeutic role of EE on the development of binge- and eating disorders and obesity. Finally, a new research avenue in this field, providing a better knowledge of EE neurochemical mechanisms, would help to develop new pharmacological modulators that mimic (environmimetics) or enhance EE positive effects (Nithianantharajah and Hannan, 2006).

## Conclusion

Preclinical studies and human research suggest that EE has a therapeutic and a protective role in drug addiction and exerts a modulatory control on excessive binge-like intake of palatable substances, including EtOH and sucrose. We know that EE also regulates excessive anxiety and compulsivity/impulsivity-like behaviors, known to be risk factors favoring the development of binge-consumption and transition to addiction. In this framework, we have proposed the working hypothesis that EE exposure might primarily modulate anxiety and impulsivity/compulsivity brain systems which, in turn, might secondarily moderate binge consumption of palatable substances preventing the progression toward addictive consumption during later stages of the addiction cycle. Future studies will further elucidate whether the observed EE therapeutic and protective effect on binge-consumption and anxiety/impulsivity/compulsivity, are causally linked.

## Author Contributions

IC and ER-O designed the conceptual framework in the manuscript and wrote the manuscript. All authors critically reviewed the content and approved the final version for publication.

## Conflict of Interest Statement

The authors declare that the research was conducted in the absence of any commercial or financial relationships that could be construed as a potential conflict of interest.
